# Transactions of Mississippi State Medical Association

**Published:** 1889-12

**Authors:** 


### BOOK NOTICES.

Transactions of the Mississippi State Medical Association. The Twenty-third Annual Session, held at Jackson, April 17th–19th, 1889.

The above-named volume contains the transactions of an allopathic medical Society; as in the latter days the papers and the discussions of most homœopathic and allopathic medical societies read so alike, we were for some time in doubt as to the status of this Society!

As so many are in these days telling us of the wonderful *advances in therapeutics* made daily by the old school, adding that now there is no difference in the practice of the two schools, hence they should be amalgamated, etc., etc., to all such prattlers we respectfully commend a careful perusal of an address upon "Medical Progress," quoted in full in this issue, taken from the above-named transactions.

